# Illicit purchasing and use of flavour accessories after the European Union menthol cigarette ban: findings from the 2020–21 ITC Netherlands Surveys

**DOI:** 10.1093/eurpub/ckad049

**Published:** 2023-04-17

**Authors:** Christina N Kyriakos, Pete Driezen, Geoffrey T Fong, Janet Chung-Hall, Andrew Hyland, Cloé Geboers, Lorraine V Craig, Marc C Willemsen, Filippos T Filippidis

**Affiliations:** Department of Primary Care and Public Health, School of Public Health, Imperial College London, London, UK; Department of Psychology, University of Waterloo, Waterloo, ON, Canada; School of Public Health Sciences, University of Waterloo, Waterloo, ON, Canada; Department of Psychology, University of Waterloo, Waterloo, ON, Canada; School of Public Health Sciences, University of Waterloo, Waterloo, ON, Canada; Ontario Institute for Cancer Research, Toronto, ON, Canada; Department of Psychology, University of Waterloo, Waterloo, ON, Canada; Health Behavior, Roswell Park Comprehensive Cancer Center, Buffalo, NY, USA; Department of Health Promotion (CAPHRI), Maastricht University, Maastricht, The Netherlands; Trimbos Institute, Netherlands Expertise Centre for Tobacco Control, Utrecht, The Netherlands; Department of Psychology, University of Waterloo, Waterloo, ON, Canada; Department of Health Promotion (CAPHRI), Maastricht University, Maastricht, The Netherlands; Trimbos Institute, Netherlands Expertise Centre for Tobacco Control, Utrecht, The Netherlands; Department of Primary Care and Public Health, School of Public Health, Imperial College London, London, UK

## Abstract

**Background:**

The 2020 European Union (EU) menthol cigarette ban increased quitting among pre-ban menthol smokers in the Netherlands, but some reported continuing to smoke menthol cigarettes. This study examined three possible explanations for post-ban menthol use—(i) illicit purchasing, (ii) use of flavour accessories and (iii) use of non-menthol replacement brands marketed for menthol smokers.

**Methods:**

Data were from the ITC Netherlands Cohort Surveys among adult smokers before the menthol ban (Wave 1: February–March 2020, *N* = 2067) and after the ban (Wave 2: September–November 2020, *N* = 1752; Wave 3: June–July 2021, *N* = 1721). Bivariate, logistic regression and generalized estimating equation model analyses were conducted on weighted data.

**Results:**

Illicit purchasing remained low from pre-ban (2.4%, 95% CI: 1.8–3.2, Wave 1) to post-ban (1.7%, 1.2–2.5%, Wave 3), with no difference between menthol and non-menthol smokers from Wave 1 to Wave 3. About 4.4% of post-ban menthol smokers last purchased their usual brand outside of the EU and 3.6% from the internet; 42.5% of post-ban menthol smokers and 4.4% of smokers overall reported using flavour accessories, with greater odds among those aged 25–39 years vs. 55+ (aOR = 3.16, *P* = 0.002). Approximately 70% of post-ban smokers who reported using a menthol brand were actually using a non-menthol replacement brand.

**Conclusions:**

There was no increase in illicit purchasing or of smuggling outside the EU among menthol and non-menthol smokers in the Netherlands 1 year after the EU menthol cigarette ban. Use of flavour accessories and non-menthol replacement brands best explain post-ban menthol use, suggesting the need to ban accessories and ensure industry compliance.

## Introduction

Flavours in tobacco can increase product appeal and attractiveness, particularly among youth, which can lead to increased smoking experimentation and progression to regular use.^1–3^ Menthol, the most popular flavour, has cooling properties, which can further mask tobacco harshness and facilitate inhalation.^4^ The World Health Organization Framework Convention on Tobacco Control calls for Parties to adopt regulations prohibiting or restricting ingredients, including flavourings.^5^ Consistent with this provision, the European Union (EU) banned characterizing flavours in boxed cigarettes and roll-your-own (RYO) tobacco in May 2016, with its application to menthol in May 2020.^6^

Evaluation studies have found that menthol cigarette bans are effective in reducing menthol use prevalence,^7^^,^^8^ and in increasing quit attempts and quitting among menthol smokers.^8–11^ A pooled analysis of data from two pre–post studies in Canada^9^^,^^10^ found that the Canadian menthol ban significantly increased quit rates among menthol smokers compared to non-menthol smokers.^11^ Similarly, a pre–post study in the Netherlands found that pre-ban menthol smokers had greater odds of making a post-ban quit attempt than non-menthol smokers.^8^ While these results suggest that EU menthol ban had a positive impact on cessation outcomes, one-third of pre-ban menthol smokers in the Netherlands reported continuing to smoke menthol cigarettes after the ban.^8^

The tobacco industry argues that menthol bans will lead to increased illicit trade and cross-border purchasing, although there has not been evidence of this in other countries.^10^^,^^12–14^ It is also plausible that smokers are getting menthol cigarettes from less regulated sources, such as the internet.

Another possible explanation for post-ban menthol use is that smokers are using legal ‘flavour accessories’ (e.g. separate capsules, RYO filters and flavour cards) to flavour unflavoured cigarettes. Market growth of these products after menthol bans in the EU, UK and Canada suggests industry exploitation of this regulatory loophole.^15–17^

Lastly, post-ban menthol use may be explained by smokers using brands that are on the post-ban market and are explicitly advertised as ‘non-menthol’, but have been marketed as a replacement or an alternative for menthol smokers.^18^^,^^19^ One such ‘non-menthol replacement’ brand, is described on an online retailer site in the Netherlands as follows, ‘Please note this article has been changed, the menthol addition is no longer in the product and will therefore taste different than before. However, an attempt has been made to match the taste as much as possible with the old product’.^20^

The aim of this pre–post cohort study of adult smokers in the Netherlands was to examine three possible explanations for self-reported post-ban menthol use—(i) illicit purchasing, (ii) use of flavour accessories and (iii) use of non-menthol replacement brands.

## Methods

### Study design

Longitudinal data came from Waves 1–3 of the International Tobacco Control (ITC) Policy Evaluation Netherlands Project with New Cohort 2020/2021 surveys, a prospective cohort study. Notably, there is considerable harmonization in design and questions between the ITC Netherlands Survey and the ITC Canada Survey, which provided data for evaluation of the Canadian menthol cigarette ban.

At the time of recruitment, respondents were adult (aged ≥18 years) cigarette smokers who smoked at least one cigarette in the last month and at least 100 cigarettes in their lifetimes. Participants were re-contacted at subsequent survey waves, regardless of their smoking status. Those who were lost to follow up at Waves 2 or 3 (*N* = 741) were replaced with new, randomly selected smokers from within the sampled areas. The Wave 1 survey was conducted from February to March 2020, before implementation of the May 2020 menthol cigarette ban (pre-ban). The Wave 2 and Wave 3 surveys were conducted from September to November 2020 and from June to July 2021, respectively, after implementation of the menthol ban (post-ban). The analytic sample was restricted to smokers who reported using cigarettes at least monthly at Wave 1 (*N* = 2067), Wave 2 (*N* = 1752) and Wave 3 (*N* = 1721), given that outcomes of interest in this study were only relevant to smokers.

Respondents were sampled from the TNS NIPObase, a database comprising more than 200 000 respondents randomly sampled from the Dutch population to participate in ongoing research by Kantar Public Netherlands. The sampling frame was designed to yield a representative random sample of smokers living in the Netherlands, within strata defined by age, gender and NUTS-1 region. Surveys were completed using computer assisted web interviews. Response rates were 57.6% at Wave 1, 58.3% at Wave 2 and 54.0% at Wave 3. Further details on the methodology can be found elsewhere.^21^

### Measures

#### Sociodemographics

Sociodemographic variables included region of residence (West, North, East and South); gender (male, female); and age group (18–24, 25–39, 40–54 and 55+). Additionally, highest level of education was categorized as low (primary education/lower pre-vocational secondary education), moderate (middle pre-vocational secondary education/secondary vocational education), high (senior general secondary education/pre-university education/higher professional education) and do not know. Monthly household income was categorized as low (<€2000), moderate (€2000–3000), high (>€3000) and do not know.^22^

#### Smoking behaviours

Smoking behaviours examined were smoking frequency (daily and non-daily); self-reported flavour of usual brand [menthol and non-menthol (plain and some other flavour)]; usual cigarette brand [factory-made (FM) and RYO]; nicotine dependence (measured by the heaviness of smoking index:^23^ low, moderate and high); and plans to quit (no plans, plans within the next 6 months and plans in the future beyond 6 months).^24^

#### Noticing and purchasing illicit cigarettes

Measures of illicit trade were assessed with the following questions: ‘Cigarettes and rolling tobacco are sometimes sold that have been smuggled, lack proper health warning labels, or do not have all government taxes paid. (1) In the last 6 months, have you seen tobacco products being sold that you thought met this description? (yes, no) (2) In the last 6 months, have you bought cigarettes or rolling tobacco in the Netherlands that might have been smuggled?’ (yes, no)

#### Purchasing behaviours

Smokers whose last purchase was their usual or current brand were asked about their purchasing behaviours. The source of last purchase was assessed using two questions: ‘Where did you last buy cigarettes or rolling tobacco for yourself—that is, from what store or seller?’ (grocery store or supermarket, bar or restaurant, duty-free shop, the internet, newsstand or kiosk, tobacconist, vending machine, gas station and other) and ‘Did you last buy your cigarettes or rolling tobacco inside or outside the Netherlands?’ (inside the Netherlands, outside the Netherlands but in the EU and outside the EU).

#### Flavour accessories

Use of flavour accessories was assessed using the question, ‘Do you add flavour(s) to your cigarettes? Select all that apply’ (yes, via flavour cards, frutasticks, filters, menthol drops and another product; no). If respondents selected one or more flavour accessories, they were also coded as ‘adding flavour via any form or via any route/mechanism’. The survey did not ask about specific flavour(s) used, except for menthol drops where it is implied.

#### Brand and menthol use validation

Respondents were asked to report their current or usual brand and variety of cigarettes. Respondents were instructed to enter the name of the brand (or part of the name) and then select their brand and variety from a pre-populated list. If their brand was not listed, they were asked to select ‘other’ and enter their brand’s name and variety. Cigarette brand names reported by smokers who said that their usual brand is menthol were coded by the research team as: (i) non-menthol, (ii) menthol and (iii) non-menthol replacement. Non-menthol replacement brands are marketed as non-menthol (i.e. alluding to compliance with the law), but suggested to be a replacement or alternative brand for menthol smokers through the insinuation that these brands have ‘menthol-like’ qualities.^19^ Brands were categorized as such if online tobacco retailers indicated that they were non-menthol and/or were described as the replacement for the banned menthol brand or an alternative for menthol smokers. Supplementary table S4 provides details of the coding sources and Supplementary table S5 displays example webpage images of how products were coded.

Among smokers reporting that their usual brand is menthol, actual menthol use was validated based on the coded brand type and/or reported use of a flavour accessory. Self-reported menthol smokers were validated to be ‘actually smoking menthol’ if they reported using a ‘menthol’ brand regardless of using a flavour accessory or if they reported using a flavour accessory regardless of the brand type. Self-reported menthol smokers were validated to be ‘actually smoking non-menthol’ if they reported using a non-menthol or replacement brand and did not report using a flavour accessory.

### Statistical analysis

Bivariate and multivariable analyses were conducted in Stata/SE 16.1 using weighted data to account for the stratified sampling design and for the oversampling of 18- to 24-year olds, with region as the stratum variable. Rescaled cross-sectional weights for Waves 1–3, respectively, were calibrated by gender and age, education and region to represent the Dutch population of smokers at the time of the survey.^21^ Covariates were identified conceptually and based on the literature,^25^^,^^26^ and were selected to be in the model based on Akaike and Bayesian information criteria, and measures used to compute sampling weights.^21^ Bivariate results are presented as percentages with 95% confidence intervals (95% CIs). Chi-square tests were conducted for bivariate comparisons between menthol and non-menthol smokers across Waves 1–3 on noticing and purchasing smuggled cigarettes. A binary generalized estimating equation regression model was fit to test the two-way interaction between menthol status and wave on purchasing smuggled cigarettes, adjusting for region, gender, age, education and flavour of usual brand by Wave interaction. Adjusted percentages and percentage point differences are presented with 95% CIs and *P*-values. A logistic regression analysis was conducted to examine correlates of using any type of flavour accessory at Wave 3, adjusting for gender, age, education and flavour of usual brand, with results presented as adjusted odds ratios (aORs) with 95% CIs.

## Results

### Baseline characteristics

Supplementary table S1 presents the baseline characteristics of smokers at recruitment, including replenishment and recontact sample (*N* = 2764) participating in Waves 1–3 of the 2020–21 ITC Netherlands Surveys.

### Pre–post ban changes in noticing and purchasing illicit cigarettes

**Figure 1:**
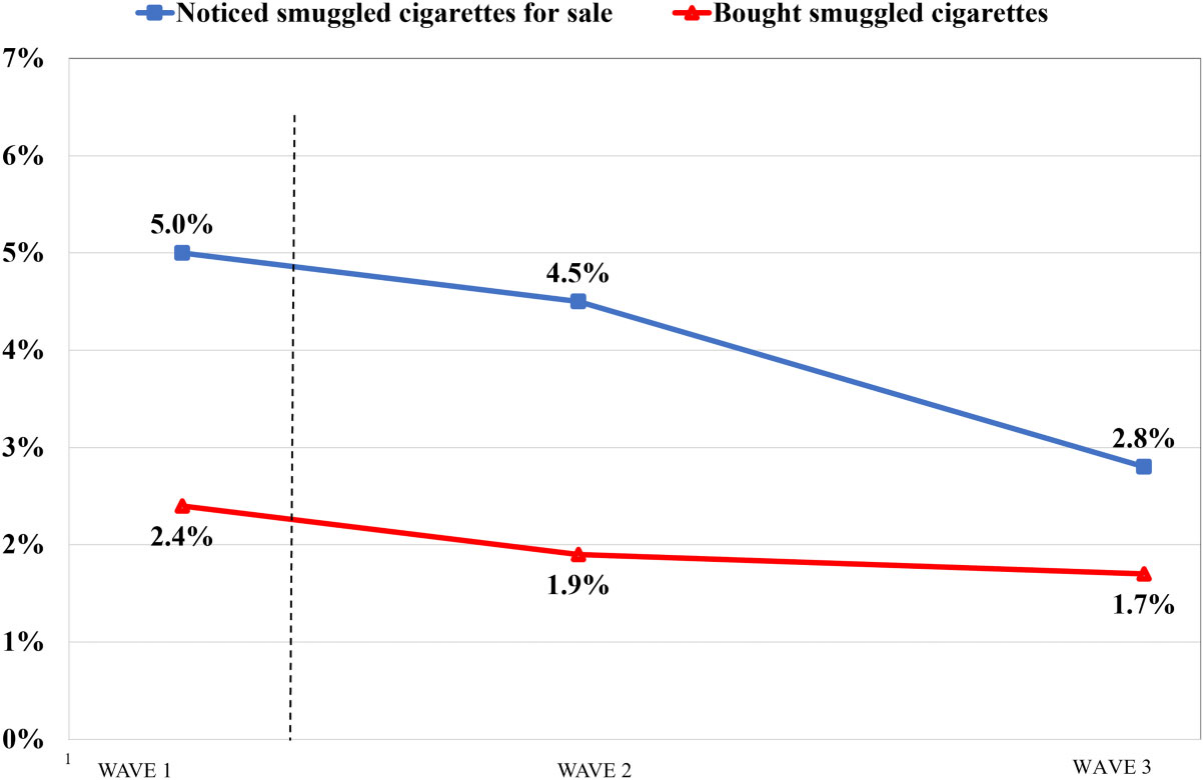
Pre- to post-ban changes in noticing^a^ and purchasing^b^ smuggled cigarettes among smokers overall, recontact and replenishment samples at Waves 1–3 of the 2020–21 ITC Netherlands Surveys,^c^ weighted. ^a^‘In the last 6 months, have you seen tobacco products being sold that you thought met this description?’ ^b^‘In the last 6 months, have you bought cigarettes or rolling tobacco in the Netherlands that might have been smuggled?’ ^c^Among at least monthly smokers; Wave 1: *N*=2067; Wave 2: *N*=1752; Wave 3: *N*=1721. *P*<0.001 for noticing smuggled cigarettes for sale between Waves 1 and 3; all other differences are not statistically significant.

As depicted in figure 1, the percentage of smokers overall reporting to have noticed smuggled cigarettes on sale within the past 6 months did not change significantly from Wave 1 (5.0%, 95% CI: 4.2–6.1) to Wave 2 (4.5%, 3.7–5.6%), and significantly decreased by Wave 3 (2.8%, 2.1–3.7%) compared to Wave 1. The percentage of smokers who reported having bought smuggled cigarettes within the past 6 months remained low across Wave 1 (2.4%, 1.8–3.2%), Wave 2 (1.9%, 1.3–2.7%) and Wave 3 (1.7%, 1.2–2.5%).

A higher proportion of menthol smokers compared to non-menthol smokers reported having bought smuggled cigarettes at Wave 2 (7.5%, 3.4–15.8% vs. 1.6%, 1.1–2.4%) compared to Wave 1 (4.6%, 2.2–9.5% vs. 2.3%, 1.7–3.1%). However, the significant difference between menthol and non-menthol smokers at Wave 2 was not sustained by Wave 3 (4.2%, 1.4–12.3% vs. 1.5%, 1.0–2.3%). Moreover, among menthol smokers, there were no significant increases in purchasing smuggled cigarettes from Wave 1 to Waves 2 or 3 (Supplementary table S2). There was also no significant interaction between wave and menthol status for purchasing smuggled cigarettes (Supplementary table S3).

### Characteristics and purchasing behaviours of post-ban menthol vs. non-menthol smokers

A higher proportion of post-ban menthol smokers (*N* = 67) at Wave 3 were female, aged 25–39 years, daily smokers, FM cigarette smokers, had low nicotine dependence (*P* < 0.001) and had high education (*P* = 0.037) than post-ban non-menthol smokers. The most common sources of menthol smokers’ last purchase of usual cigarette brand at Wave 3 were the grocery store/supermarket (41.1%), gas station (32.2%) and tobacconist (20.3%), with fewer reporting purchasing from the internet (3.6%), duty-free shop (1.4%) and ‘other’ source (1.3%). There were no significant differences in last purchase source between menthol and non-menthol smokers. Most menthol smokers bought their last pack of cigarettes from inside the Netherlands (80.7%), while some purchased outside of the Netherlands but inside the EU (14.9%). A higher proportion of menthol smokers bought their last pack of cigarettes outside of the EU compared to non-menthol smokers (4.4% vs. 0.5%, *P* < 0.001) (table 1).

**Table 1:** Characteristics and purchasing behaviours of post-ban menthol vs. non-menthol smokers, recontact and replenishment sample at Wave 3 of the 2020–21 ITC Netherlands Surveys^a^, weighted (*N* = 1752)

	Menthol smoker (*N* = 67)		Non-menthol smoker (*N* = 1685)		Comparison
Variable	*n*	% (95% CI)	*n*	% (95% CI)	*P*-value
Gender					
Female	46	72.5 (60.2–82.1)	739	43.0 (40.6–45.5)	<0.001
Male	18	27.5 (17.9–39.7)	908	56.9 (54.5–59.3)	
Age group (years)					
18–24	10	16.3 (9.0–27.8)	206	11.4 (10.0–13.0)	<0.001
25–39	31	44.6 (32.9–57.0)	406	22.5 (20.6–24.5)	
40–54	17	30.1 (19.7–43.1)	481	29.5 (27.3–31.8)	
55+	6	8.9 (4.0–18.8)	554	36.5 (34.1–39.0)	
Education					
Low	19	31.7 (21.2–44.5)	590	38.0 (35.6–40.4)	0.037
Moderate	22	32.5 (22.2–44.9)	698	40.2 (37.8–42.6)	
High	23	35.7 (24.8–48.3)	350	21.8 (19.8–24.0)	
Smoking frequency					
Daily smoker	43	67.5 (54.9–77.9)	1454	88.5 (86.9–90.0)	<0.001
Non-daily smoker	21	32.5 (22.1–45.0)	193	11.4 (10.0–13.1)	
Heaviness of smoking index					
Low (0–1)	32	52.3 (39.8–64.6)	512	31.3 (29.0–33.6)	<0.001
Moderate (2–4)	30	47.7 (35.4–60.2)	998	61.6 (59.1–63.9)	
High (5–6)	0	0.0	111	7.1 (5.9–8.5)	
Plans to quit within next 6 months					
Yes	15	25.7 (15.9–38.7)	376	28.8 (26.3–31.3)	0.621
No	40	74.3 (61.3–84.1)	922	71.2 (68.7–73.7)	
Usual brand FM or RYO					
Factory-made (FM)	62	97.2 (89.5–99.3)	1047	62.2 (59.8–64.6)	<0.001
Roll-your-own tobacco (RYO)	2	2.8 (0.7–10.5)	594	37.8 (35.4–40.2)	
Purchased smuggled cigarettes^b^					
Yes	3	4.2 (1.4–12.3)	24	1.5 (1.0–2.3)	0.084
No	60	95.8 (87.7–98.6)	1568	98.5 (97.7–99.0)	
Last purchase source^c^					
Grocery store or supermarket	24	41.1 (29.2–54.1)	903	56.5 (54.0–58.9)	0.251
Bar or restaurant	0	0.0	4	0.2 (0.1–0.6)	
Duty-free shop	1	1.4 (0.2–9.5)	15	0.9 (0.5–1.5)	
The internet	2	3.6 (0.9–13.8)	21	1.4 (0.9–2.1)	
Newsstand or kiosk	0	0.0	27	1.7 (1.2–2.5)	
Tobacconist	13	20.3 (12.0–32.3)	267	17.3 (15.5–19.3)	
Vending machine	0	0.0	4	0.2 (0.1–0.6)	
Gas station	20	32.2 (21.7–45.0)	350	21.1 (19.1–23.1)	
Other	1	1.3 (0.2–8.5)	12	0.7 (0.4–1.3)	
Last bought cigarettes from where^d^					
Inside the Netherlands	51	80.7 (69.3–88.6)	1403	86.6 (84.9–88.1)	<0.001
Outside the Netherlands, but inside EU	10	14.9 (8.1–25.7)	225	12.9 (11.4–14.7)	
Outside of the EU	3	4.4 (1.4–12.8)	8	0.5 (0.2–1.0)	
Use flavour accessories^e^					
ANY type	27	42.5 (30.8–55.1)	52	3.0 (2.3–4.0)	<0.001
Flavour cards	4	5.6 (2.1–14.1)	7	0.4 (0.2–0.9)	<0.001
Frutasticks	2	2.8 (0.7–10.7)	7	0.4 (0.2–0.8)	0.004
Filters	15	23.9 (14.8–36.2)	28	1.6 (1.1–2.3)	<0.001
Menthol drops	8	12.6 (6.3–23.6)	9	0.5 (0.3–1.0)	<0.001
Another product	5	7.4 (3.1–16.6)	13	0.8 (0.4–1.4)	<0.001

a Among at least monthly smokers at Wave 3 (post-ban): June–July 2021.

b ‘In the last 6 months, have you bought cigarettes or rolling tobacco in the Netherlands that might have been smuggled?’

c ‘Where did you last buy cigarettes or rolling tobacco for yourself—that is, from what store or seller?’

d ‘Did you last buy your cigarettes or rolling tobacco inside or outside the Netherlands?’

e Use flavour accessories: ‘Do you add flavour(s) to your cigarettes? Select all that apply’.

### Use of flavour accessories at post-ban

Among the entire sample of smokers at Wave 3 (*N* = 1721), 4.4% reported adding any flavour to their cigarettes. Those aged 25–39 years had greater odds of using a flavour accessory compared to those aged 55+ years (aOR = 3.16, 95% CI: 1.53–6.52, *P* = 0.002). Menthol smokers were much more likely to use flavour accessories than non-menthol smokers (42.5% vs. 3.0%, aOR = 17.33, 9.33–32.19, *P* < 0.001) (table 2).

**Table 2:** Correlates of using flavour accessories^a^ at post-ban among smokers at Wave 3 of the 2020–21 ITC Netherlands Surveys^b^, recontact and replenishment sample, weighted (*N* = 1721)

Variable	*n*	% (95% CI)	**aOR (95% CI)** ^c^	*P*-value
Overall	79	4.4 (3.5–5.5)		
Gender				
Female	47	6.0 (4.5–7.9)	1.43 (0.86–2.38)	0.165
Male	32	3.2 (2.2–4.5)	1.00	
Age group (years)				
18–24	13	5.8 (3.4–9.8)	2.24 (0.92–5.44)	0.074
25–39	34	7.7 (5.5–10.7)	3.16 (1.53–6.52)	0.002
40–54	21	4.3 (2.8–6.5)	1.98 (0.93–4.23)	0.078
55+	11	1.9 (1.0–3.4)	1.00	
Education				
High	22	5.5 (3.6–8.3)	0.79 (0.41–1.52)	0.479
Moderate	27	3.6 (2.4–5.2)	0.66 (0.36–1.21)	0.177
Low	30	4.8 (3.3–6.8)	1.00	
Flavour of usual brand				
Menthol	27	42.5 (30.8–55.1)	17.33 (9.33–32.19)	<0.001
Non-menthol	52	3.0 (2.3–4.0)	1.00	

a ‘Do you add flavour(s) to your cigarettes? Select all that apply’ (yes, via flavour cards, frutasticks, filters, menthol drops and/or another product; no).

b Among at least monthly smokers at Wave 3 (post-ban): June–July 2021.

c Logistic regression model adjusted for gender, age, education and flavour of usual brand at Wave 3.

### Brand and menthol use validation

**Figure 2:**
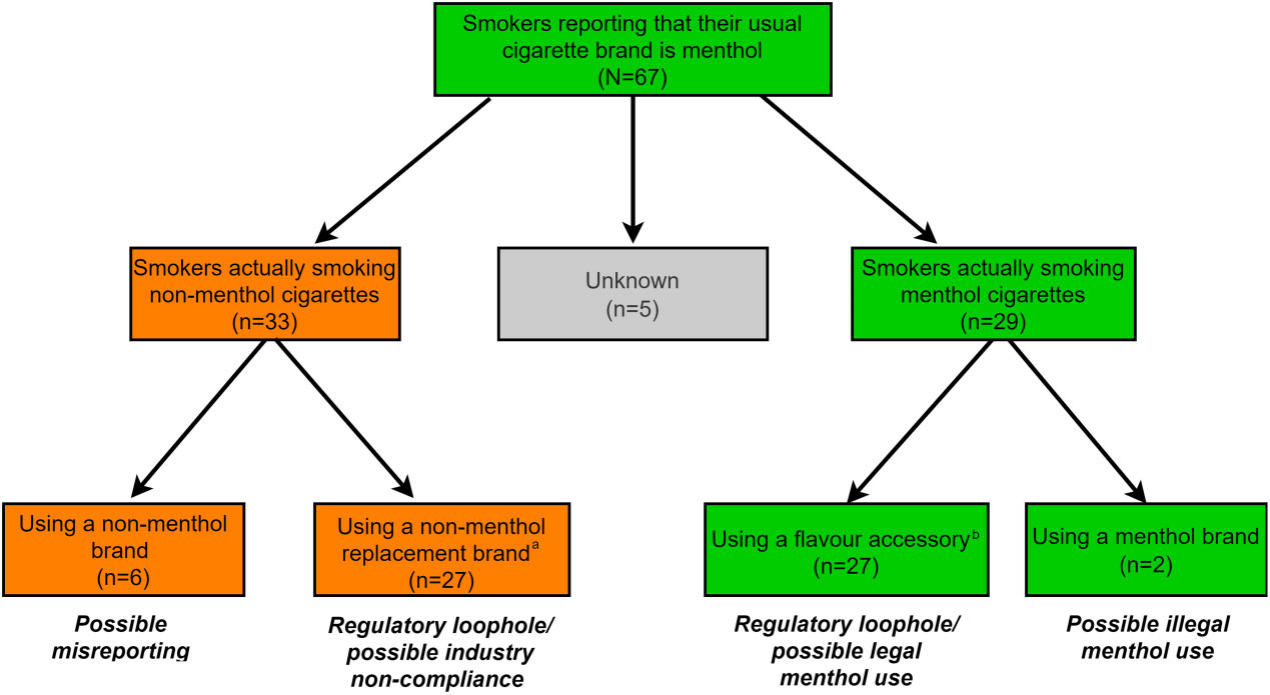
Menthol use validation analysis of post-ban smokers who self-reported that their usual brand is menthol (*N* = 67) ^a^Non-menthol replacement brands are marketed as non-menthol (i.e. alluding to compliance with the law), but suggested to be a replacement or alternative brand for menthol smokers through the insinuation that these brands have ‘menthol-like’ qualities. ^b^The question on flavour accessory use did not specify ‘menthol’ for all product options, and therefore it is possible smokers could be referring to a flavour other than menthol.

Among the 23 usual/current brands reported by smokers who self-reported that their usual brand is menthol at Wave 3 (*N* = 67 smokers), 14 brands were coded by the research team as non-menthol (*n* = 21 smokers), 1 brand as menthol (*n* = 2 smokers), 8 brands as non-menthol replacement (*n* = 48 smokers) and the brand was unknown for *n* = 6 smokers (Supplementary table S4). As displayed in figure 2, among the 67 self-reported menthol smokers, 29 were ‘smokers actually smoking menthol’, 33 were ‘smokers actually smoking non-menthol’ and menthol use was ‘unknown’ for 5 smokers. Among the smokers ‘actually smoking menthol’, almost all were considered menthol smokers based on using legal flavour accessories, with only two smokers using an actual menthol brand.

## Discussion

In this cohort study of adult smokers in the Netherlands, we found that few smokers reported noticing smuggled cigarettes for sale and purchasing smuggled cigarettes prior to and following implementation of the EU menthol cigarette ban. Further, there was no significant difference between menthol and non-menthol smokers in purchasing illicit cigarettes before the ban and 1 year after the ban. Purchasing outside of the EU and from less regulated retail environments, such as the internet, was uncommon among menthol smokers. The use of flavour accessories was low among smokers overall, although younger adults and menthol smokers were more likely to use them than older adults and non-menthol smokers, respectively. Most smokers who reported that they were smoking menthol cigarettes at post-ban were using legal flavour accessories and/or were using a non-menthol replacement alternative brand. Only two smokers reported using an actual menthol brand.

Our findings that the EU menthol ban did not increase illicit purchasing refutes the most common industry argument against menthol bans^27^ and are consistent with studies from Canada^10^^,^^13^^,^^14^ and England.^12^ Although one study concluded that the Canadian menthol ban resulted in evasion behaviours due to increased cigarette purchasing on First Nations reserves,^28^ the outcome measure in that study was purchasing of all cigarettes on First Nations reserves rather than just menthol cigarettes, diluting those brands that might show an increase in purchasing after the menthol ban (menthols) with those that would not be affected (non-menthols), with the latter outweighing the former by about 20–1, given the 5% share of menthol cigarettes in Canada prior to the ban.^14^

Upon examination of other purchasing behaviours that may help to explain where post-ban menthol users obtained their menthol cigarettes, we found a low percentage (4.4%) of reported purchasing from outside of the EU, where menthol cigarettes may be legal. However, this was significantly higher than non-menthol smokers. While it does not appear that purchasing outside of the EU is driving post-ban menthol use, menthol smokers may be more inclined to purchase menthol cigarettes while abroad. A study in England also found low levels of cross-border purchasing after the menthol ban.^12^ One of the possible avenues through which illicit cigarettes could be purchased is the internet, given the inherent complexities of regulating online retailers.^29^ Evidence from the USA suggests that the internet was a common source for purchasing illicit flavoured cigarettes after a national ban on characterizing flavours (except menthol) in cigarettes.^30^ However, in our study, a negligible minority of post-ban menthol smokers purchased from the internet. Rather, the majority last purchased their usual brand from common tobacco retailers, which was no different than non-menthol smokers.

Another possible explanation for post-ban menthol smoking is the use of legal flavour accessories to flavour unflavoured cigarettes. Approximately 40% of smokers who reported that their usual cigarette brand was menthol also reported using flavour accessories. While use of flavour accessories was not measured prior to the ban in our study, it is likely that use of these products increased from before to after the ban, as found in Canada.^25^ Another study in the Netherlands examining Nielsen sales data reported a 10% increase in the volume of flavour accessories from 1 year before to 1 year after the menthol ban.^26^ There are reports of significant efforts by the tobacco industry to point retailers and consumers to flavour accessories, as well as other non-cigarette menthol products, as an alternative to banned menthol cigarettes.^15^^,^^16^ The high proportion of menthol smokers using flavour accessories may be an indication that the tobacco industry was somewhat successful in exploiting this legislative loophole.^15^^,^^16^ While the menthol ban has been found to be effective in increasing cessation in the Netherlands,^8^ impact may be further maximized by banning flavour accessories.

Our finding that only 4.4% of smokers overall used a flavour accessory is lower, but generally consistent, with the few studies that have examined this.^25^^,^^26^ A study conducted in the Netherlands in July 2021 (the same as Wave 3 in our study) found that 11% of adult cigarette smokers were currently using a flavour accessory.^26^^,^^31^ It is possible that the latter study’s inclusion of additional varieties of accessory products for respondents to select from (i.e. stone/stick, rolling paper with taste) compared to our study may partly explain differences in estimates.^26^ The breadth of such products on the market, which continues to proliferate adds to challenges of estimating population-level use of flavour accessories. Similar to what has been found for regular menthol cigarettes,^4^ use of flavour accessories was highest among young adults in our study, consistent with other studies.^25^^,^^26^ In addition to their novel features these products are available in a variety of flavours, which make them appealing to youth and young adults.^26^ Prevalence of flavour accessories, particularly among young people, warrants continued monitoring. Future research should explore how these products are marketed to consumers.

Our finding that 70% of self-reported menthol smokers reported using a non-menthol replacement brand suggests the possibility that some smokers may still be perceiving these products as being mentholated. There is evidence that the tobacco industry is exploiting the regulatory ambiguity of banning menthol as a ‘characterising flavour’, rather than as an additive.^19^^,^^32^ Indeed, sensory and chemical testing indicate that multiple products on the EU market contain a characterizing flavour.^33^ Moreover, given that there is evidence that menthol can still achieve cooling effects even at levels below the threshold of what would be detectable as a characterizing flavour, a complete ban on menthol additives (and its analogues), as is done in Canada, may better achieve public health impact.^34^^,^^35^

Limitations of this study must be considered. While the overall sample size was large, the sub-group sample sizes for menthol use and illicit purchasing were small, which may have reduced statistical power to detect differences in pre–post illicit purchasing rates between menthol and non-menthol smokers. However, pre–post trends in the overall sample suggest that the proportion of smokers who noticed smuggled cigarettes for sale significantly decreased. Misclassification bias of outcome measures could have also occurred due to the design of some survey questions. For instance, purchase source was limited to smokers’ last purchase of their usual brand and therefore may not fully capture the extent to which respondents may have purchased from specific sources. Moreover, incidental purchasing of illicit products may have been overlooked. However, focussing on last purchase rather than a longer time frame would have reduced recall bias. Moreover, our measurement of flavour accessory use did not distinguish between flavour accessories that were menthol and those that were nor did it list the entire inventory of flavour accessories on the market. There is evidence though that menthol is the most commonly used accessory flavour in the Netherlands.^26^^,^^31^ As previously mentioned, misclassification of brands as menthol or non-menthol may have occurred since respondents selected their brand from a pre-populated list. Nevertheless, this study is strengthened by its use of a prospective cohort study design and its quasi-experimental design that allows for comparison of those affected by the ban (i.e. menthol smokers) to a comparison group who were not affected by the ban (i.e. non-menthol smokers).

This study has significant policy implications. Findings support the growing evidence that menthol bans do not appear to increase illicit purchasing; policymakers should be wary of industry rhetoric used to oppose the implementation of such bans, which have demonstrated significant public health benefits.^8^^,^^10^^,^^11^^,^^36^ Additionally, while post-ban menthol cigarette purchasing did not seem to be driven by cross-border purchasing or the internet in the Netherlands during this period, menthol cigarette purchasing should continue to be monitored, particularly among youth and in other EU countries. A draft decision on an amendment of the Tobacco and Tobacco Products Decree in the Netherlands would prohibit internet sales of all tobacco products from 1 July 2023 onwards.^37^ Post-ban menthol use appears to be best explained by reported use of flavour accessories or use of non-menthol replacement brands that are marketed for menthol smokers, both of which are regulatory loopholes of the EU menthol ban that have been exploited by the tobacco industry.^32^ Policymakers should consider expanding flavour regulations to cover flavour accessories, as Belgium, Denmark and Lithuania have done,^26^ and to adopt a complete ban on menthol additives and its analogues, as Germany has done.^35^ Finally, the US Food and Drug Administration’s proposed rule to ban menthol in cigarettes^38^ invited comments on whether menthol flavour accessories should also be prohibited. In our study, we observed a high prevalence of such flavour accessories, highlighting the need to ban such products to eliminate one method of enabling menthol smokers to continue smoke menthol cigarettes rather than quitting.

## Supplementary Material

ckad049_Supplementary_Data

## Data Availability

In each country participating in the International Tobacco Control Policy Evaluation (ITC) Project, the data are jointly owned by the lead researcher(s) in that country and the ITC Project at the University of Waterloo. Data from the ITC Project are available to approved researchers 2 years after the date of issuance of cleaned datasets by the ITC Data Management Centre. Researchers interested in using ITC data are required to apply for approval by submitting an International Tobacco Control Data Repository (ITCDR) request application and subsequently to sign an ITCDR Data Usage Agreement. The criteria for data usage approval and the contents of the Data Usage Agreement are described online (http://www.itcproject.org). Key points Illicit purchasing did not increase after the EU menthol cigarette ban, with no significant difference between menthol and non-menthol smokers who purchased illicit cigarettes before the ban and 1 year after the ban. A small minority of post-ban menthol smokers last purchased their usual brand outside of the EU and from less regulated sources, such as the internet, with no differences in purchasing sources between menthol and non-menthol smokers. Overall, only 4.4% of smokers reported using flavour accessories; however, use was higher among younger adults and among self-reported post-ban menthol smokers. Nearly all self-reported post-ban menthol smokers reported using either flavour accessories and/or a non-menthol replacement brand, making these the most likely explanations of post-ban menthol use. Impact of the EU menthol ban may be maximized by also banning flavour accessories and menthol as an additive, and by ensuring industry compliance.
