# Supplementary material for: Illicit purchasing and use of flavour accessories after the European Union menthol cigarette ban: findings from the 2020–21 ITC Netherlands Surveys
Source: Eur J Public Health. 2023 Apr 17;33(4):619–26. doi: 10.1093/eurpub/ckad049 (PMC10393478; doi:10.1093/eurpub/ckad049)
Supplement: ckad049_Supplementary_Data [file ckad049_supplementary_data.docx]

**SUPPLEMENTARY TABLES**

**Supplementary Table 1. Baseline characteristics of** **smokers at recruitment, Waves 1-3 of the 2020-2021 ITC Netherlands Surveys, replenishment and recontact sample, weighted (N=2764)**

| **Variable** | **n** | **%** | **95%CI** |
| --- | --- | --- | --- |
| **Wave of recruitment** |  |  |  |
| Wave 1 | 2067 | 75.1 | (73.4, 76.7) |
| Wave 2 | 268 | 9.5 | (8.4, 10.6) |
| Wave 3 | 429 | 15.5 | (14.1, 16.9) |
| **Region** |  |  |  |
| West | 1159 | 46.5 | (46.2, 46.8) |
| North | 334 | 11.8 | (11.6, 12.0) |
| East | 576 | 20.4 | (20.2, 20.6) |
| South | 695 | 21.3 | (21.1, 21.5) |
| **Gender** |  |  |  |
| Male | 1474 | 55.1 | (53.2, 56.9) |
| Female | 1290 | 44.9 | (43.1, 46.8) |
| **Age group (years)** |  |  |  |
| 18-24 | 478 | 15.8 | (14.5, 17.2) |
| 25-39 | 811 | 26.4 | (24.8, 28.0) |
| 40-54 | 712 | 27.3 | (25.6, 29.0) |
| 55+ | 763 | 30.5 | (28.8, 32.4) |
| **Household income** |  |  |  |
| Low | 635 | 22.8 | (21.3, 24.5) |
| Moderate | 517 | 18.8 | (17.3, 20.3) |
| High | 974 | 35.3 | (33.5, 37.1) |
| Not stated | 638 | 23.1 | (21.6, 24.8) |
| **Education** |  |  |  |
| Low | 1015 | 38.8 | (37.0, 40.7) |
| Moderate | 1178 | 40.5 | (38.7, 42.4) |
| High | 548 | 20.6 | (19.1, 22.3) |
| **Smoking frequency** |  |  |  |
| Non-daily smoker | 432 | 15.3 | (14.0, 16.7) |
| Daily smoker | 2332 | 84.7 | (83.3, 86.0) |
| **Usual brand FM or RYO** |  |  |  |
| Factory-made (FM) | 1901 | 67.5 | (65.7, 69.3) |
| Roll-your-own tobacco (RYO) | 851 | 32.5 | (30.7, 34.3) |
| **Heaviness of smoking index (HSI)** |  |  |  |
| Low (0-1) | 930 | 33.8 | (32.0, 35.6) |
| Moderate (2-4) | 1636 | 60.2 | (58.3, 62.1) |
| High (5-6) | 159 | 6.0 | (5.1, 7.0) |
| **Plans to quit within next six months** |  |  |  |
| Yes | 1455 | 66.6 | (64.6, 68.6) |
| No | 751 | 33.4 | (31.4, 35.4) |
| **Flavour of usual brand** |  |  |  |
| Menthol | 201 | 7.1 | (6.2, 8.1) |
| Non-menthol (plain or other flavour) | 2549 | 92.9 | (91.9, 93.8) |

**Supplementary Table 2. Percentage of smokers who purchased cigarettes that may have been smuggled^1^ before and after the menthol ban, recontact and replenishment samples at Waves 1-3 of the 2020-2021 ITC Netherlands Surveys**^2^**, weighted, unadjusted**

|  | **Purchased cigarettes that may have been smuggled** | | | | | | | | | | | |
| --- | --- | --- | --- | --- | --- | --- | --- | --- | --- | --- | --- | --- |
|  | **Pre-ban (Wave 1)** | | |  | **Post-ban (Wave 2)** | | | | **Post-ban (Wave 3)** | | |  |
|  | **n** | **%** | **95%CI** | **p-value** | **n** | **%** | **95%CI** |  | **n** | **%** | **95%CI** | **p-value** |
| **Flavour of usual brand** |  |  |  |  |  |  |  |  |  |  |  |  |
| Menthol | 7 | 4.6 | (2.2, 9.5) | 0.075 | 6 | 7.5 | (3.4, 15.8) | <0.001 | 3 | 4.2 | (1.4, 12.3) | 0.084 |
| Non-menthol | 43 | 2.3 | (1.7, 3.1) |  | 26 | 1.6 | (1.1, 2.4) |  | 24 | 1.5 | (1.0, 2.30) | |

^1^ *“In the last six months, have you bought cigarettes or rolling tobacco in the Netherlands that might have been smuggled?”*

^2^ Among at least monthly smokers; Wave 1 (pre-ban): February to March 2020 (N=2067); Menthol ban implemented: May 2020; Wave 2 (post-ban): September to November 2020 (N=1752); Wave 3 (post-ban): June to July 2021 (N=1721)

**Supplementary Table 3. Predictors of purchasing smuggled cigarettes^1^, recontact and replenishment samples at Waves 1-3 of the 2020-2021 ITC Netherlands Surveys^2^, weighted, adjusted**

| **Variable** | **Adj %*** | **95%CI** | **PP diff*** | **95%CI** | **p-value** |
| --- | --- | --- | --- | --- | --- |
| **Interaction model †** |  |  |  |  |  |
| Wave 1 (pre-ban) | 2.4 | (1.7, 3.0) | Ref |  |  |
| Wave 2 (post-ban) | 1.9 | (1.3, 2.6) | -0.5 | (-1.2, 2.8) | 0.221 |
| Wave 3 (post-ban) | 1.8 | (1.2, 2.4) | -0.6 | (-1.4, 0.2) | 0.156 |
| Non-menthol | 1.8 | (1.4, 2.3) | Ref |  |  |
| Menthol | 5.6 | (2.5, 8.8) | 3.8 | (0.6, 7.0) | 0.021 |
| Wave 1 x Non-menthol | 2.2 | (1.6, 2.9) | Ref |  |  |
| Wave 2 x Menthol | 7.2 | (1.8, 12.6) | 2.9 | (-2.8, 8.6) | 0.321 |
| Wave 3 x Menthol | 4.8 | (0.1, 8.6) | 0.4 | (-3.7, 4.5) | 0.841 |

^1^ *“In the last six months, have you bought cigarettes or rolling tobacco in the Netherlands that might have been smuggled?”*

^2^ Among at least monthly smokers; Wave 1 (pre-ban): February to March 2020 (N=2067); Menthol ban implemented: May 2020; Wave 2 (post-ban): September to November 2020 (N=1752); Wave 3 (post-ban): June to July 2021 (N=1721)

**†** GEE model adjusted for region, gender, age, education, and flavour of usual brand by Wave interaction

* Post-estimation margins commands in Stata

Adj= Adjusted; GEE= Generalised estimating equation; PP diff= Percentage point differences, Ref= Reference group

**Supplementary Table 4. Brand analysis of reported brands used by smokers who self-reported that their usual brand is menthol at post-ban by whether or not the respondents reported using a flavour accessory, Wave 3 of the ITC 2020-2021 Netherlands Surveys (N=67)**

| Brand name | Brand type | Brand coding source | Use flavour accessories (N=40) | Do NOT use flavour accessories (N=27) | Total |
| --- | --- | --- | --- | --- | --- |
| Camel Activate Blue | Non-menthol replacement | ^[[1]](#footnote-2)^ ^[[2]](#footnote-3)^ ^[[3]](#footnote-4)^ ^[[4]](#footnote-5)^ | 0 | 5 | 5 |
| Camel Activate Green | Non-menthol replacement | a b c d | 2 | 5 | 7 |
| Camel Activate White | Non-menthol replacement | a b c d | 0 | 4 | 4 |
| Camel Blue | Non-menthol | a b c d | 0 | 1 | 1 |
| Camel Yellow | Non-menthol | a b c | 1 | 0 | 1 |
| Elixyr Blue | Non-menthol | ^[[5]](#footnote-6)^ | 2 | 0 | 2 |
| Elixyr Green/Elixyr Plus | Non-menthol replacement | c ^[[6]](#footnote-7)^ | 9 | 2 | 11 |
| Elixyr Red | Non-menthol | b c | 2 | 0 | 2 |
| JPS Red | Non-menthol | a c d | 1 | 0 | 1 |
| JPS XL Blue | Non-menthol | a d | 1 | 0 | 1 |
| L&M Blue Label | Non-menthol | a d | 1 | 0 | 1 |
| L&M Forward | Non-menthol replacement | a b d | 0 | 2 | 2 |
| L&M Red Label | Non-menthol | a b c d | 0 | 1 | 1 |
| Lucky Strike Blend (roll-your-own tobacco) | Non-menthol | a b c | 0 | 1 | 1 |
| Lucky Strike Ice Cold | Non-menthol replacement | a b | 0 | 1 | 1 |
| Marlboro Beyond Blue | Menthol | e | 1 | 1 | 2 |
| Marlboro Black Beyond | Non-menthol | d | 1 | 0 | 1 |
| Marlboro Fuse Beyond/Marlboro Beyond Green | Non-menthol replacement | d ^[[7]](#footnote-8)^ | 0 | 6 | 6 |
| Marlboro Gold | Non-menthol | a b c d | 4 | 1 | 5 |
| Marlboro Green/Marlboro Bright | Non-menthol replacement | b ^[[8]](#footnote-9)^ | 0 | 2 | 2 |
| Pall Mall Blue | Non-menthol | a b c | 0 | 2 | 2 |
| Winston Blue | Non-menthol | a b c d | 1 | 0 | 1 |
| Other- Signature American Blend “met een klik" | Non-menthol + accessory | ^[[9]](#footnote-10)^ | 0 | 1 | 1 |
| Other- No brand listed | - | - | 1 | 1 | 2 |
| Don't know | - | - | 0 | 4 | 4 |

**Supplementary Table 5. Webpage screenshots from online tobacco retailers in the Netherlands exemplifying of how non-menthol replacement cigarette brands vs non-menthol cigarette brands are indicated (as of 20^th^ October 2022)**

| **Online Retailer** | **Non-menthol replacement brand** | **Non-menthol brand** |
| --- | --- | --- |
| (a) Dutch Tobacconist by Hartman Cigars  <https://www.dutchtobacconist.nl/> | 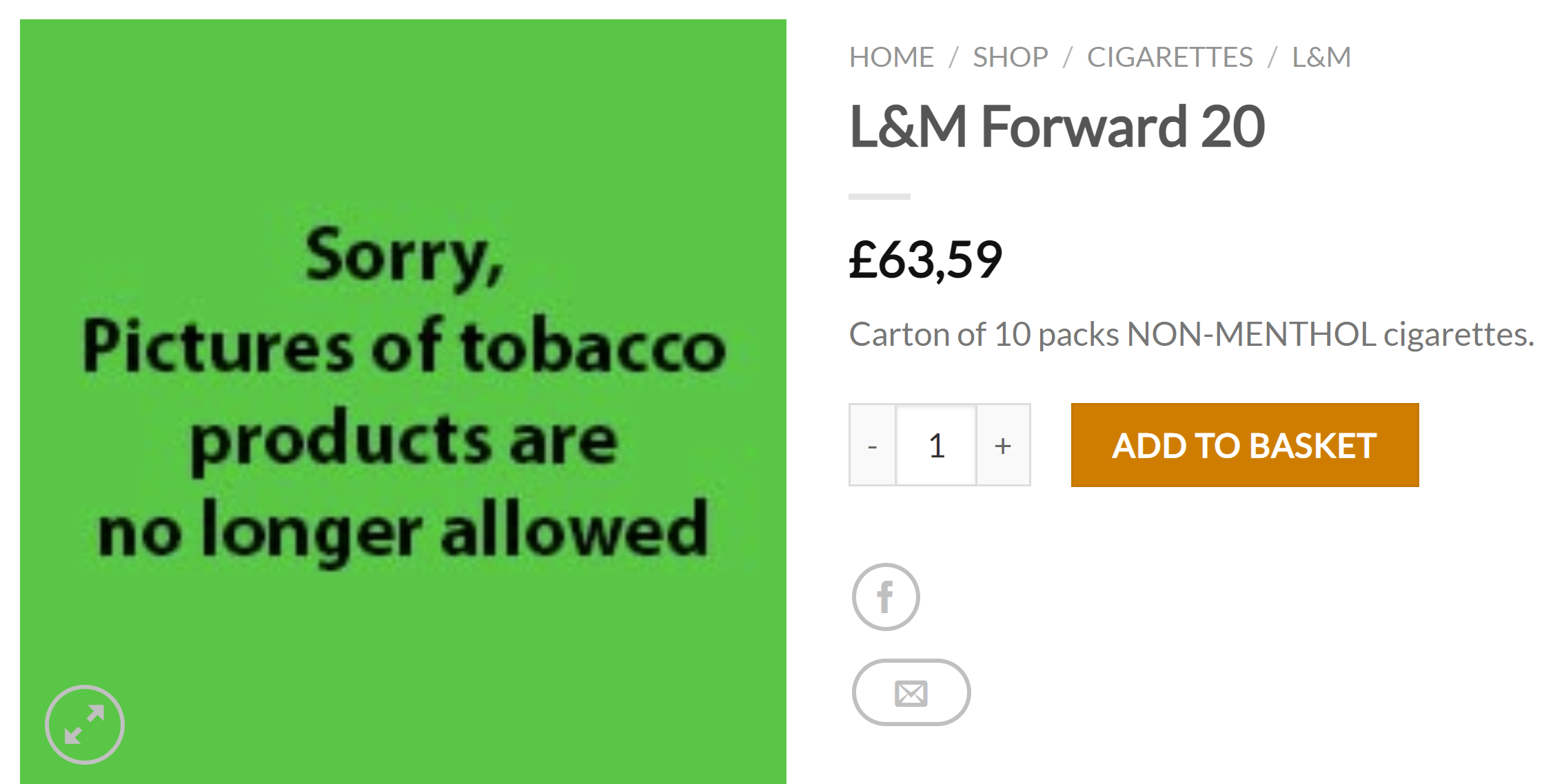  ***“NON-MENTHOL cigarettes”*** | 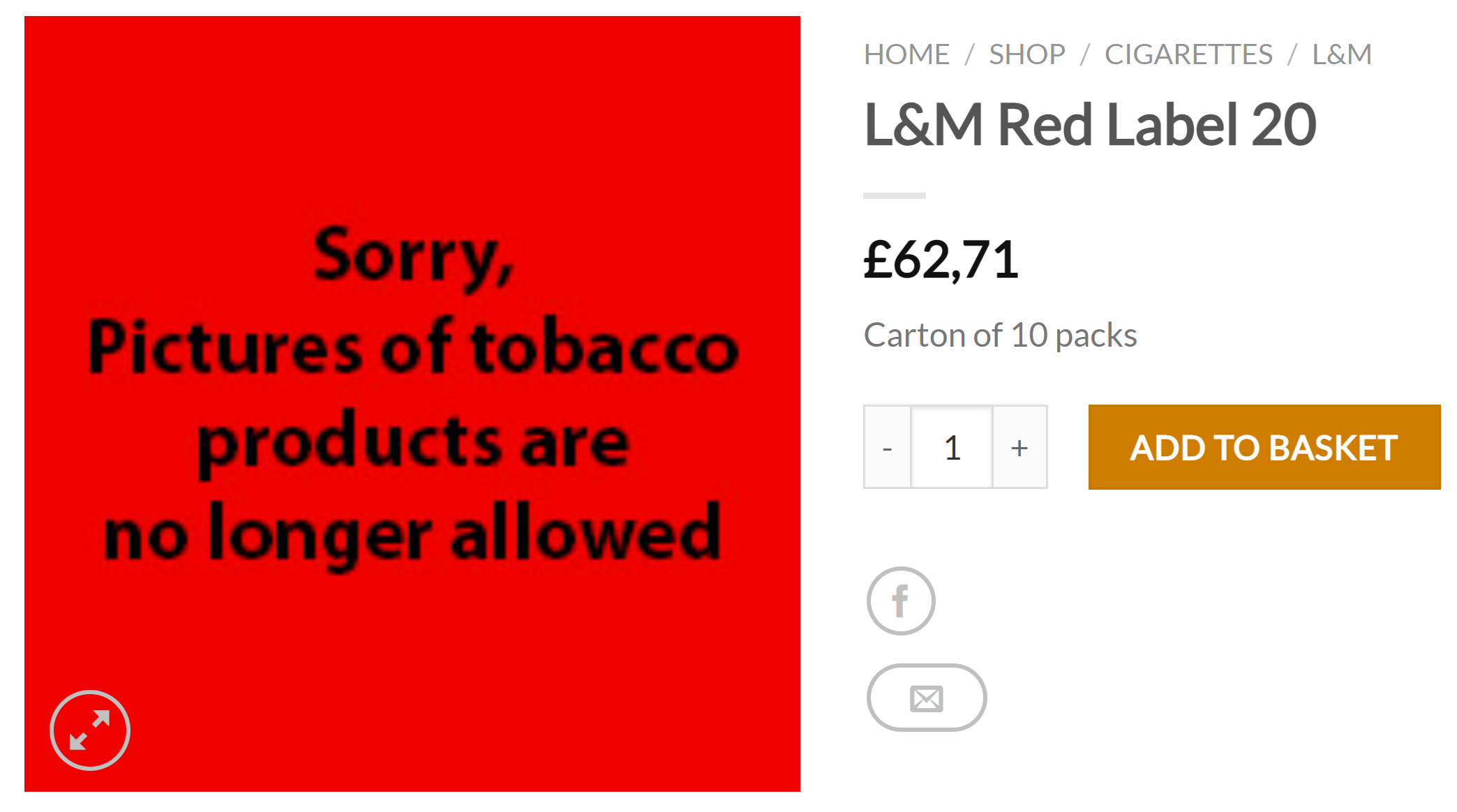 |
| (b) Rookwaren-Online  <https://www.rookwaren-online.nl/en_GB/> | 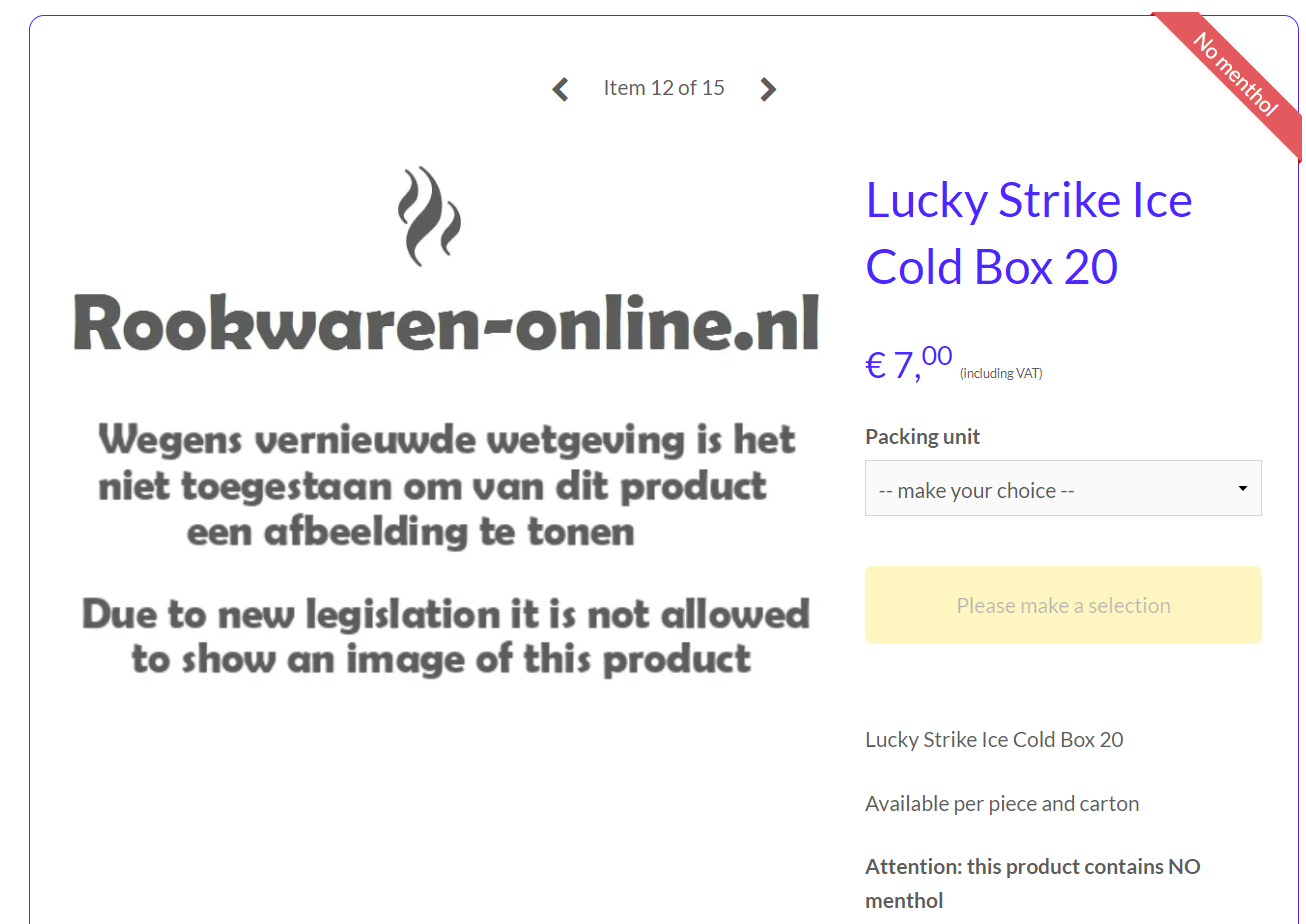  ***“This product contains NO menthol”*** | 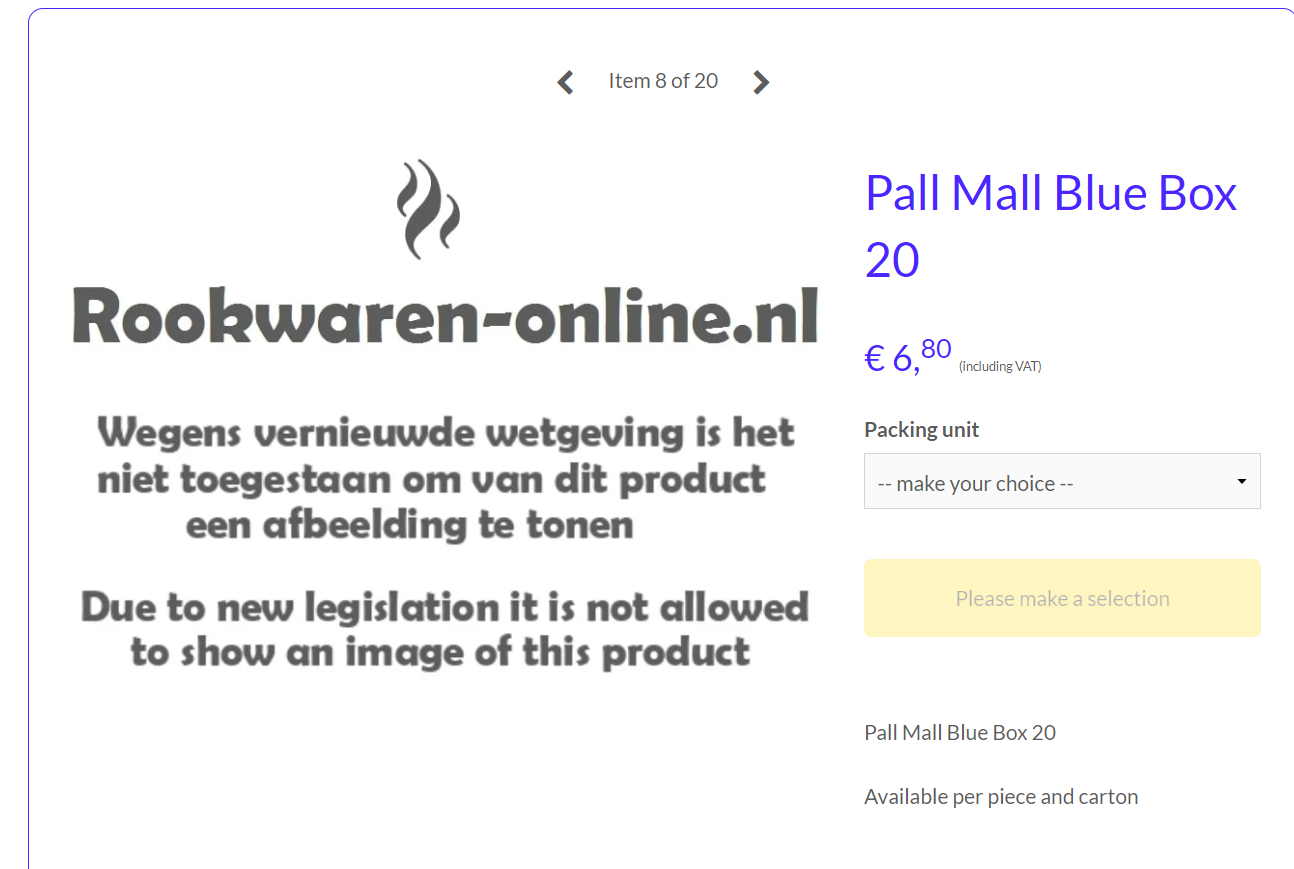 |
| (c) Robrijkers  <https://www.robrijkers.nl/> | 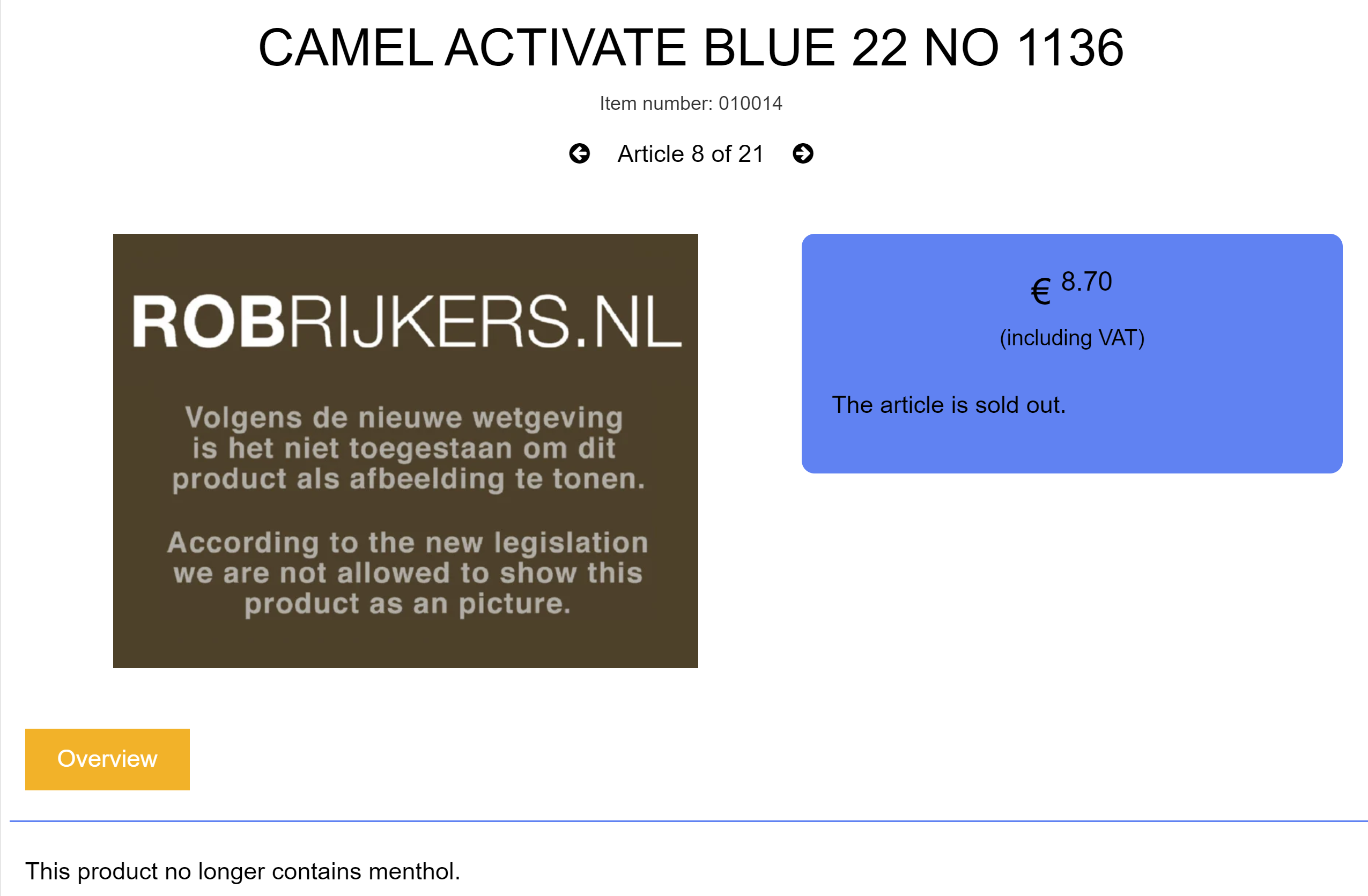  ***“This product no longer contains menthol”*** | 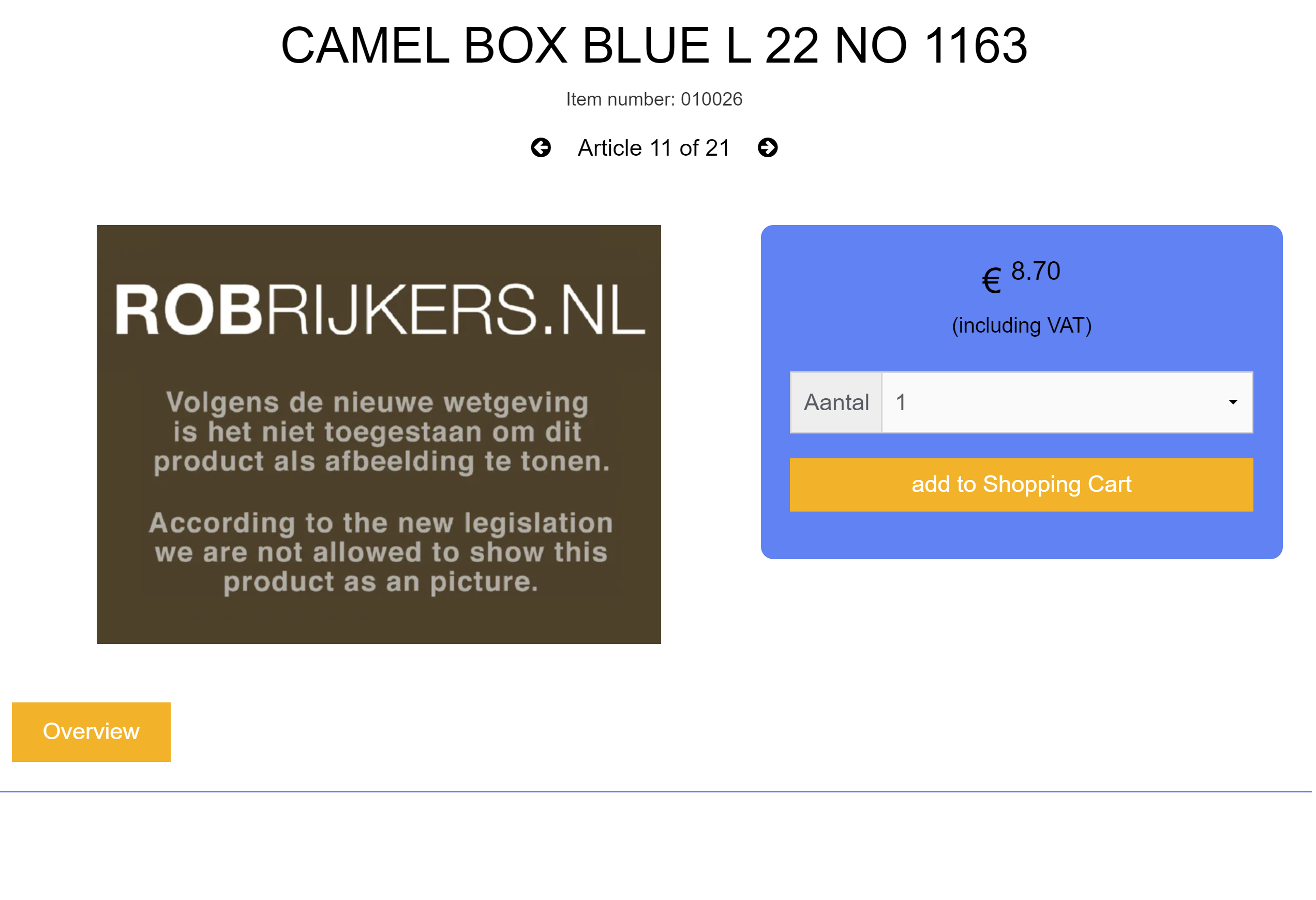 |
| (d) Hartman Cigars  <http://www.hartmancigars.nl/> | 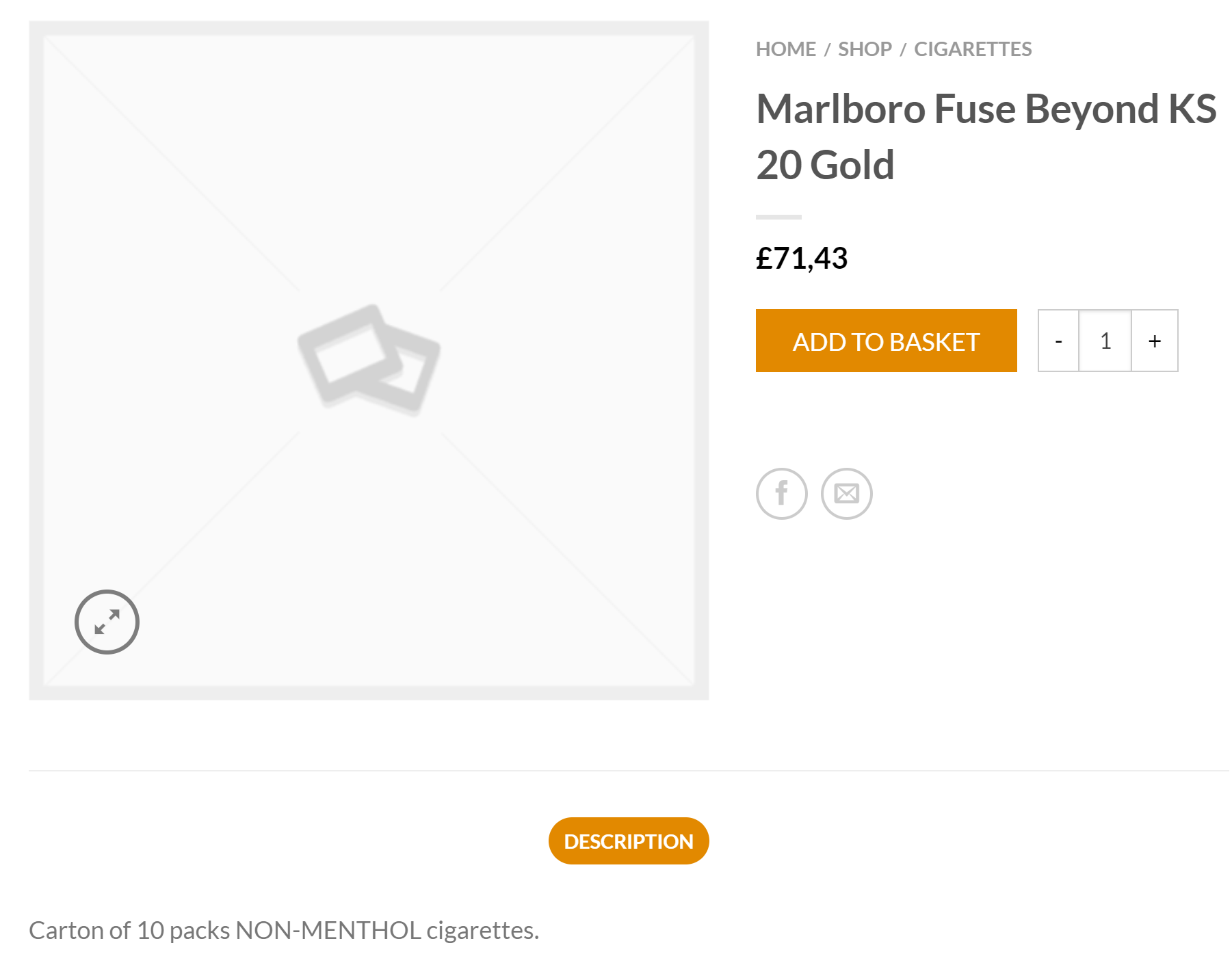  ***“NON-MENTHOL cigarettes”*** | 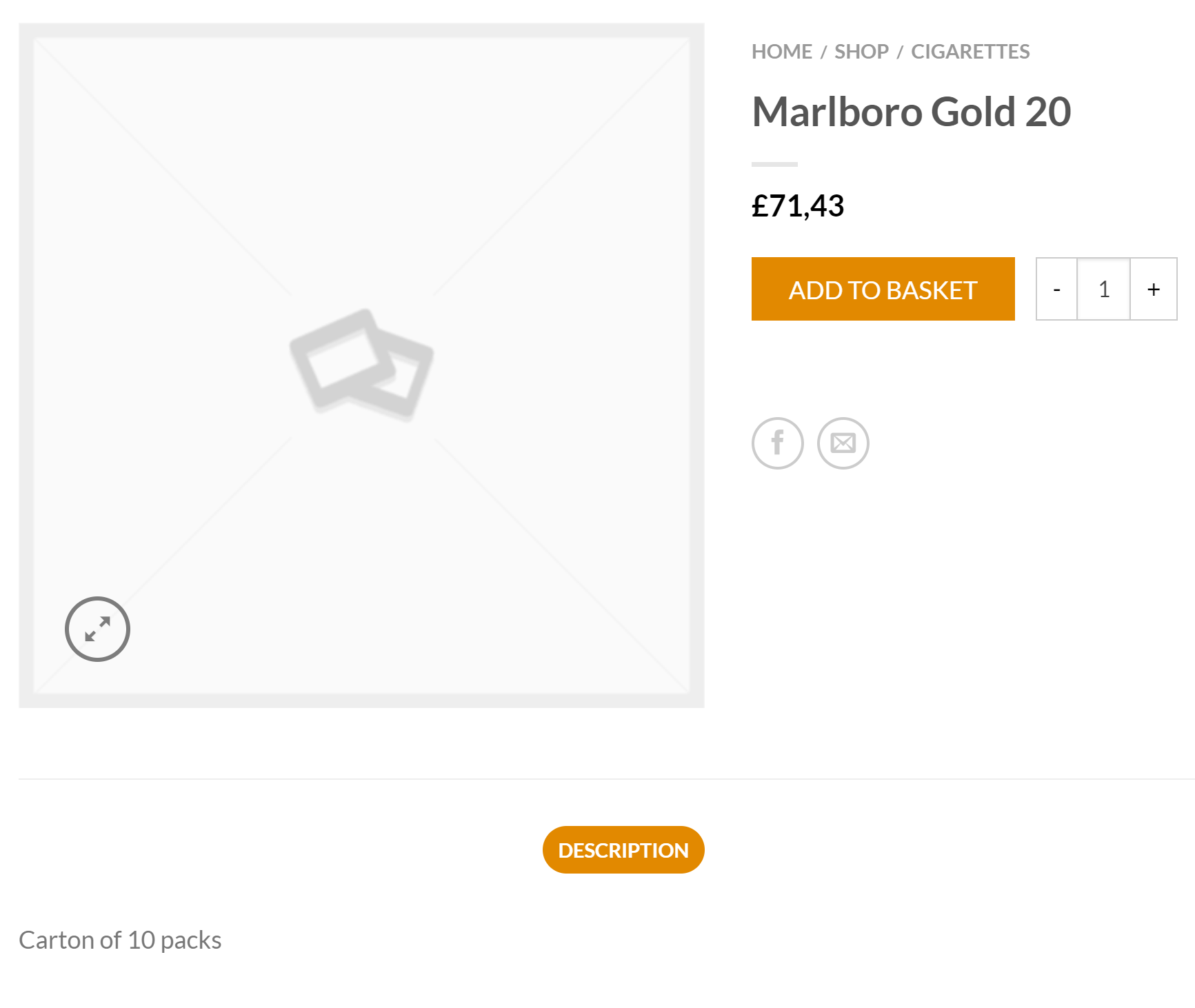 |

1. Dutch Tobacconist by Hartman Cigars; non-menthol replacement if labelled as "Non-menthol cigarettes", non-menthol if not labelled <https://www.dutchtobacconist.nl/> [↑](#footnote-ref-2)
2. Rookwaren-Online; non-menthol replacement if labelled as "This product contains no menthol”; non-menthol if not labelled; <https://www.rookwaren-online.nl/> [↑](#footnote-ref-3)
3. Robrijkers; non-menthol replacement if labelled as "This product no longer contains menthol"; non-menthol if not labelled. <https://www.robrijkers.nl/> [↑](#footnote-ref-4)
4. Hartman Cigars; non-menthol replacement if labelled as "Non-menthol cigarettes", non-menthol if not labelled <http://www.hartmancigars.nl/> [↑](#footnote-ref-5)
5. National Institute for Public Health and the Environment (RIVM) “Toevoegingen tabaksproducten”, published tobacco products ingredients data from 2011-2015 reported by tobacco manufacturers to the European Union Common Entry Gate. “Menthol” indicated in parentheses of product name; “Non-menthol” if menthol not specified” in parentheses or in brand name. <https://www.rivm.nl/toevoegingentabaksproducten/products.html> [↑](#footnote-ref-6)
6. Elixyr Plus is advertised as a normal cigarette to be used with a flavour accessory: “This is a normal Elixyr cigarette with a 6mm hole in the filter part, into which a 6mm menthol filter is inserted, the result is that you have a comparable menthol experience as a menthol cigarette, pay attention because here is only the filter of menthol and not, as with a menthol cigarette, the tobacco that has been processed.” <https://www.sigarenmagazijnhethoekje.nl/Elixyr-Plus-sigaretten-> [↑](#footnote-ref-7)
7. Marlboro Fuse Beyond/Marlboro Beyond Green (<https://primeradedriehoek.nl/product/marlboro-fuse-beyond/>) is advertised as having a “fresh flavour” (<https://www.rokado.nl/tabak/sigaretten/marlboro-beyond-green-detail.html>) [↑](#footnote-ref-8)
8. Marlboro Green/Marlboro Bright is advertised as having an “altered taste” (<https://www.rokado.nl/tabak/sigaretten/marlboro-bright-20-11247-detail.html>) and that "the menthol addition is no longer in the product and will therefore taste different than before. However, an attempt has been made to match the taste as much as possible with the old product.” (<https://www.sigaren.shop/marlboro-green-bright-sigaretten-bestellen.html>) [↑](#footnote-ref-9)
9. “Klik” in Dutch means “click”, and therefore respondent may be referring to adding the flavour accessory “click ball/bead” [↑](#footnote-ref-10)
